# Shaping Macromolecules for Sensing Applications—From Polymer Hydrogels to Foldamers

**DOI:** 10.3390/polym14030580

**Published:** 2022-01-31

**Authors:** Simone Giuseppe Giuffrida, Weronika Forysiak, Pawel Cwynar, Roza Szweda

**Affiliations:** 1 Łukasiewicz Research Network—PORT Polish Center for Technology Development, ul. Stabłowicka 147, 54-066 Wrocław, Poland; Giuseppe.Giuffrida@port.lukasiewicz.gov.pl (S.G.G.); Weronika.Forysiak@port.lukasiewicz.gov.pl (W.F.); Pawel.Cwynar@port.lukasiewicz.gov.pl (P.C.); 2 Faculty of Chemistry, University of Wrocław, F. Joliot-Curie, 50-383 Wrocław, Poland; 3 Faculty of Chemistry, Wrocław University of Science and Technology, Wybrzeże Wyspiańskiego 27, 50-370 Wrocław, Poland

**Keywords:** polymer gels, hydrogels, foldamers, sensing, biosensing

## Abstract

Sensors are tools for detecting, recognizing, and recording signals from the surrounding environment. They provide measurable information on chemical or physical changes, and thus are widely used in diagnosis, environment monitoring, food quality checks, or process control. Polymers are versatile materials that find a broad range of applications in sensory devices for the biomedical sector and beyond. Sensory materials are expected to exhibit a measurable change of properties in the presence of an analyte or a stimulus, characterized by high sensitivity and selectivity of the signal. Signal parameters can be tuned by material features connected with the restriction of macromolecule shape by crosslinking or folding. Gels are crosslinked, three-dimensional networks that can form cavities of different sizes and forms, which can be adapted to trap particular analytes. A higher level of structural control can be achieved by foldamers, which are macromolecules that can attain well-defined conformation in solution. By increasing control over the three-dimensional structure, we can improve the selectivity of polymer materials, which is one of the crucial requirements for sensors. Here, we discuss various examples of polymer gels and foldamer-based sensor systems. We have classified and described applied polymer materials and used sensing techniques. Finally, we deliberated the necessity and potential of further exploration of the field towards the increased selectivity of sensory devices.

## 1. Introduction

Sensing is a very important division in applied sciences, present in several sectors such as diagnosis and treatment [1,2], environment monitoring [3], processes control [4], and food quality analysis [5]. Quickly developing medicine requires tools for rapid, cost-effective, and reliable diagnosis to provide proper patient treatment. This need has distinctly become evident in the crisis of the COVID-19 outbreak, generating a huge necessity for widespread testing. Besides medicine, effective sensors are needed for security reasons, e.g., to be able to detect illegal explosives. Moreover, facing climate changes, environmental monitoring has become crucial to mitigate harmful effects. However, there is a lack of appropriate tools. The World Health Organization (WHO) has warned about the importance of water quality monitoring to control the level of bioactive substances. However, suitable methods have not been developed so far [6]. The need for sensors is huge and unsatisfied due to the insufficiency of affordable and practical devices suitable for common use.

A typical sensor is a device able to receive signals and stimuli from the environment and deliver output data about changes in its surroundings [7,8]. In other words, the function of a sensor is to detect events or changes in the environment and provide information about these alterations. The crucial component of the sensor is a probe material, sensitive to particular changes (e.g., temperature, presence of an analyte, pH), able to transduce information to a readable physical signal [9,10]. Usually, the probe material determines the sensing parameters, such as detection limit, selectivity, specificity, and appropriate detection method. Several ways to transduce such signals have been established and classified according to various detection techniques. Among them, we can distinguish optical sensing (absorbance, reflectance, luminescence, fluorescence, index of refraction, opto-thermal and scattering effects); electrochemical sensing (voltammetry, amperometry, potentiometry and field-effect); mass sensing (piezoelectricity and surface acoustic wave effects); thermometric sensing (heat effects derived from chemical reaction or absorption); and radiation sensing (based on the absorbance of radioactive species) [11]. Apart from this classification, sensors can be classified by their probe elements (such as polymer, ionophore, enzymes, antigens/antibodies, cell, protein and membrane receptors, tissues, oligonucleotides, specific ligands, etc.) and by the sensed analyte (such as glucose, DNA, enzymes, toxins, drugs, etc.). Sensing and biosensing have progressively become crucial for various applications, e.g., process regulation, quality control, environmental monitoring, and diagnosis.

Polymers are versatile materials that represent a broad range of properties and stimuli-responsiveness, thus very attractive for sensors construction [12,13,14,15,16]. These materials can be adapted to specific tasks through their synthesis or modification [17,18,19] and may be used as solid membranes, gels, nanoparticles, or thin films [20,21,22,23]. Polymer gels are three-dimensional networks that can form cavities of different sizes and shapes, which can be adapted to trap particular analytes. Those features have been used to obtain various sensory materials [7,23,24,25,26]. Gels can be formed by covalent or non-covalent bonds, e.g., ionic and hydrogen bonds [27]. Gel materials in sensing can be generally classified into hydrogels formed by natural [28,29,30,31] or synthetic [16,32,33] polymers [22,25]. Gels coupled with other types of materials, e.g., gold nanoparticles [34,35,36,37,38] and quantum dots [39,40], can be distinguished as a hybrid group of hydrogel materials [31]. An interesting materials in the context of sensing applications are foldamers [41,42,43]. These materials can attain particular secondary structures, similarly to natural peptides and proteins, thus they are very attractive as sensory materials. The foldamer-based sensors have been described in a separate section of this review article. Another type of crosslinked polymer material widely applied in sensing is molecularly imprinted polymers [44]. The principle of molecular imprinting is based on the self-assembly of a crosslinked polymer matrix in the presence of a template molecule. In these materials, monomers should be carefully chosen to present a proper functional group to interact covalently or non-covalently with a template molecule. The assembled structure is covalently stabilized by a polymerization reaction and the template analyte is removed, leaving a cavity fitted to the analyte. Molecularly imprinted polymers are a subject of several review articles [45,46,47,48] and are not discussed in this article.

Here, we discuss various examples of polymer gel-based sensor systems. This article briefly classifies gels with an emphasis on the chemical structure based on examples from the last decade. We discuss applied detection techniques and sensing parameters used for polymer gel systems. Finally, we deliberated the necessity and possibility of further exploration of the field towards the increased selectivity of polymer materials. We mention some recent advances in the field of gels, along with unsolved issues, and suggest possible solutions.

## 2. Hydrogel Materials in Sensing

Polymeric hydrogels are viscoelastic networks resembling deformable solid-state materials made of hydrophilic polymers, but due to the crosslinked structure they are not soluble in water, yet highly absorbent [49,50]. The water-uptake capability of the hydrogel provides a suitable environment for biomolecules. Thus, they maintain long-term bioactivity [51,52]. Hydrogel structures can be formed by covalent or non-covalent interactions, forming interconnections called crosslinks, among the polymer backbone parts [53,54]. The nature of the crosslinks may be either chemical, via covalent bonds, or physical, via weak interactions such as coordinative, electrostatic, hydrophobic and dipole-dipole or chain entanglements between the segments of the network [23,55]. The capacity to retain a high content of water imparts to the hydrogels the ability to swell and be soft materials stable in aqueous media [56,57]. The properties of the hydrogels can be easily modulated by structure modification to induce responsiveness to external stimuli [58,59] or sensitivity to particular analytes [60]. Hydrogels can undergo volume-phase transitions when they are exposed to a stimulus or molecular interactions with the analyte. Such phase transition results in changing hydrogel properties, e.g., swelling, collapse, or solution-to-gel transition, transparency, and conductivity, which can be exploited to construct sensor probes [7,24,56]. They can change their properties under external inputs, e.g., the presence of particular ions [61] or bioactive molecules, pH [62,63,64], temperature [65], light radiation [66], electric [67,68] or magnetic [69,70] fields, etc. [71]. For example, pH-sensitive hydrogels contain carboxylic or amine groups in their chains which can respond to the change of pH and ionic strength in the medium [63]. Depending on pH, the hydrogel displays reversible swelling/de-swelling properties [26,72]. Another method to derivatize the hydrogels is to attach (bio)molecules that can recognize selectively particular ligands. For instance, Wei et al. developed a microfluidic device that incorporates an aptamer-based hydrogel for the detection of cocaine [56,73]. Hydrogels, thanks to the aforementioned characteristics, such as the ability to retain molecules of water and swell in media, softness, and ease of derivatization, have gained interest as transducer materials in sensing and biosensing technologies [7,9,12].

Examples of hydrogel sensing and biosensing applications are listed in Table 1 and Table 2 and classified according to the detection method. The detection methods used in gel-based sensors can be considered into two main categories: electrochemical or optical techniques. Hydrogels for optical detection methods contain fluorophores or chromophores in their chains, which interact through various mechanisms with the target molecules. The interaction with the analyte alters measured optical property, e.g., color [74], absorbance [75], fluorescence emission [76], or volume [77]. For example, Haldar and Lee [78] developed a polymeric chemosensor for the detection of Hg^2+^ ions in aqueous media. N,N-dimethylacrylamide hydrogel chains were decorated by thiosemicarbazide modified BODIPY fluorogenic groups. Hydrogel itself did not display fluorescence. However, in the presence of Hg^2+^ ions, it showed a noticeable fluorescence emission enhancement at 545 nm. The change in optical properties was due to the restricted isomerization of the C=N bond of the thiosemicarbazide unit caused by complexation with Hg^2+^ ions. Besides devices with optical detection, polymer gels are used as electrochemical (bio)sensors. They are built into a three-electrode electrochemical cell, consisting of a working electrode (WE), a counter or an auxiliary electrode, and a reference electrode. Different materials are used as WEs, e.g., glassy carbon electrodes, carbon paste electrodes, pencil graphite electrodes, indium tin oxide electrodes, pyrolytic graphite electrodes, gold or platinum electrodes, and carbon or gold screen-printed electrodes [12,79]. The WE presents the electroconductive materials which are responsible for biorecognition event with the targeted species, causing the change of the electrochemical signal, recorded as impedance, current, or voltage. To achieve conductive properties, gels are doped with conducting materials, e.g., polymers [80], carbon materials [81], metal nanoparticles [82], or quantum dots [39]. For example, Çevik et al. [79] developed a label-free biosensor for detecting the prostate-specific antigen (tPSA) in serum samples. A conductive layer of poly(amidoamine) dendrimers crosslinked by glutaraldehyde, containing ferrocene units was assembled on gold electrode. The capture antibody for tPSA was covalently attached to the dendrimer terminal groups. Once the antibody captures the tPSA present in the serum, ferrocene undergoes a redox transition. The change in the redox state of ferrocene induces an alteration in the voltage, read as a differential pulse voltammetry signal [79].

Polymeric materials used for sensors are divided into natural hydrogels, derived from natural polymers, e.g., proteins (collagen [76,80,83], elastin [84,85], gelatin [86,87,88], albumin [29,89]), polysaccharides (hyaluronic acid [90], alginate [91,92,93,94,95], chitosan [82,96,97,98,99,100,101], cellulose [74]), and synthetic hydrogels [39,102,103,104,105,106], based on laboratory-made polymers. Hydrogels in sensing applications are often used as a hybrid material, blend of polymers or in composition with inorganic materials, e.g., graphene, graphene oxide, or silica [31]. In hybrid hydrogel systems, each of the components has a function [107]. For instance, carbon additives provide material conductivity that enables the application of amperometric detection techniques. Hydrogels can be used in diverse forms, suitable for final applications, e.g., hydrogel films, self-standing substrate, or nano and microparticles [55,88,92,108].

### 2.1. Sensors Based on Natural Hydrogels

Natural hydrogels are derived from naturally occurring polymers that are cross-linked by covalent or supramolecular bonds [109]. The classification of the natural hydrogels includes two main groups, based on their derivation. The first group is represented by protein-based materials [76,80,83,84,85,86,87,88,89] which include collagen, elastin, fibrin, gelatin, and skin fibroin. The second group is constituted of hydrogels derived from polysaccharides [74,82,90,91,92,93,96,97,98] such as glycosaminoglycans, alginate and chitosan. Such hydrogels are suitable for most biological applications attributable to their biocompatibility and bioactivity. If needed, the properties of natural hydrogels can be adjusted by chemical modification [28] or the preparation of composite polymers with synthetic hydrogels [100]. Natural hydrogels have been used for the sensing of glucose [80,93,97,98,100], dopamine [83], antioxidants [82], pH [74,88,89], explosives [39,85,86,87,96], biomarkers [76,84,90,91,92], and body signals [29,30] using electrochemical and optical detection methods (Table 1).

**Table 1:** Natural hydrogel-based sensors.

Hydrogel	Sensing	Analyte	Characteristics	Ref.
**Electrochemical Methods**				
**Polyacrylic acid-lignosulfonate-alginate-Ca^2+^**	Resistance	Strain	Resistance changes vs. time when monitoring different body joints motions, responsive performance up to 500 cycles. Compression stress—835 kPa Tensile fracture stress—357 kPa Stretching strain—1144%	[28]
**BSA crosslinked by cysteine disulfide bridges**	Amperometry	Physiological signals	Electrocardiography (ECG) for heart activity, electroencephalography (EEG) for brain activity, and electrooculography (EOG) for eye activity, conductivity = 5.3 mS cm^−1^	[29]
**Chitosan/cationic guar gum**	Amperometry	Human body motions	0.296 kPa pressure sensitivity when pressure was lower than 1.25 kP	[30]
**Methacrylated-collagen, polypyrrole and glucose oxidase**	Amperometry	Glucose	LOD = 2 mM, PBS buffer (pH = 7.4) LOD ≅ 200 mM in porcine meat Linear range: 0–4 mM High selectivity in vivo	[80]
**Collagen from grass carp skin, graphene oxide and aptamer**	Linear Sweep Voltammetry (LSV)	Dopamine	LOD = 0.75 nM, PBS buffer (pH = 7) Linear range: 1–1000 nM	[83]
**Alginate copper oxide with glucose oxidase**	Amperometry	Glucose	LOD = 1.6 µM in human serum Linear ranges: 0.04–3 mM and 4–35 mM Sensitivity: 30.443 and 7.025 µA mM^−1^ cm^−2^ Selectivity among ascorbic acid, uric acid, acetaminophen and phenylalanine	[93]
**Chitosan crosslinked with silver ions**	Linear Sweep Voltammetry (LSV)	Antioxidants (ascorbic acid)	Linear range: 0.04 µM–36 µM 0.1 mM H_2_O_2_ solution (pH = 4.5) Selectivity among glucose and sucrose	[82]
**Chitosan crosslinked with genipin, amino-derived osmium redox complex and glucose oxidase**	Amperometry	Glucose	Linear range: ~0.1–20 mM in PBS buffer (pH = 7)	[97]
**Laponite-chitosan with lactate oxidase on glassy carbon electrode**	Amperometry	*L*-lactate	LOD = 3.8 µM in alcoholic beverages Sensitivity: 0.326 A M^−1^ cm^−2^ LOQ = 12.6 µM Linear range: 10–70 µM	[98]
**Chitosan, oxidized** **dextran, and CeO_2_/MnO_2_ hollow nanospheres**	Amperometry	Glucose	LOD = 32.4 μM in PBS buffer (pH = 7.4) Sensitivity: 176 μA mM^−1^ cm^−2^ Linear range: 1–111 mM	[99]
**3-aminopropyltriethoxysilane/chitosan with glucose oxidase**	Amperometry	Glucose	LOD = 0.2 µM, 0.10 M PBS buffer (pH = 7.0) Linear range: 0.2 µM–8.2mM and 0.2 µM–5.5 mM Sensitivity: 69.5 and 65 µA mM^−1^ cm^−2^	[100]
**Chitosan-carbon nanotubes (Chitosan-CNTs)**	Cyclic Voltammetry (CV)	Dopamine	LOD = 2.00 vs. 1.00 µmol L^−1^ Sensitivity: 3.00 vs. 0.01 µA L µmol^−1^ (CNT loading 1.75% vs. 1%) Linear range: 0–10 µM for both in 300 μmol L^−1^ uric acid solution	[101]
**Pectin/reduced graphene oxide**	Cyclic Voltammetry (CV), Linear Sweep Voltammetry (LSV)	Dopamine, Paracetamol	LOD = 1.5 nM (Dopamine) LOQ = 0.4 nM (Dopamine) Linear range (LSV): 0.003–90.206 µM (Dopamine) LOD = 1.8 nM (Paracetamol) LOQ = 0.6 nM (Paracetamol) Linear range (LSV): 0.003–91.04 µM (Paracetamol) Both were performer in PBS (pH = 7.0)	[110]
**Optical methods**				
**Pyrophosphate ion-alginate with carbon dots and Cu^2+^**	Fluorescence	Alkaline phosphatase (ALP)	LOD = 0.55 mU/mL Linear range: 0–100 mU/mL λ_em_ = 513 nm gel-sol transition	[61]
**Collagen-lysyl oxidase**	Fluorescence/Imaging	Biomarkers Lysyl oxidase	Turn-on fluorescence probe Extracellular matrix Before binding—ϕ_F_ = 0.09 λ_abs_ = 360 nm, λ_em_ = 395 nm After binding—ϕ_F_ = 0.89 λ_abs_ = 310 nm, λ_em_ = 455 nm	[76]
**Human elastin-like polypeptide and bilirubin-binding protein UnaG**	Fluorescence	Biomarkers, detection of bilirubin	Linear range: 0–100 nM in PBS buffer pH = 7.4 cell culture media; λ_em_ = 528 nm, λ_exc_ = 485 nm	[84]
**Silk/Elastin-like recombinamers with fluorescent proteins (SELR-FPs)**	Fluorescence	protein eqFP650	λ_ex_ = 475 nm, λ_em_ = 636 nm FRET pairs–fluorescent proteins AcEGFP and eqFP650, potential use as a biosensor	[85]
**Gelatin methacryloyl**	Tactile sensing	Pressure change	LOD = 0.1 Pa Sensitivity: 0.19 kPa^−1^ Durability up to 3000 cycles Suitable for wearable biosensing application	[86]
**Gelatin-tannic acid**	Volumetric	Mechanical change	Elongation 1600% Self-healing—0.65 s Self-healing efficiency—95% (Hydrogel combined with a resistor)	[87]
**Gelatin crosslinked with carbon dots**	Photoluminescence (PL)	pH	Increasing pH in range 3–10, Linear range: 5–7 pH PL quenching at λ_em_ = 431 nm, λ_ex_ = 350 nm	[88]
**Human serum albumin (HSA)-manganese complex**	Magnetic resonance imaging (MRI)	pH	HSA-Mn^2+^ hydrogel capsule for in situ monitoring of gastric pH	[89]
**Hyaluronic acid (HA)**	Fluorescence	Hyaluronidase (HAse)	FRET-based quenching mechanism (FITC-donor, AuNPs–acceptor). Binding to HAse prevents FRET fluorescence quenching. LOD = 0.14 U/mL Linear range: 0.5–100 U/mL Selectivity among different ions (NaCl, KCl, MgSO_4_, CaCl_2_, small molecules (glutathione, glucose, glutamine and ascorbic acid), BSA, and enzymes (alkaline phosphatase, trypsin, papain).	[90]
**Alginate crosslinked** **with Cu^2+^**	Fluorescence Immunoassay	Alkaline phosphatase (ALP)	LOD = 0.24 ng/mL (serum) Linear range: ~0–2 ng/mL Hepatis B surface antigen (HBsAg) Selectivity among Na^+^, K^+^, HAS, lysozyme, thrombin, glucose oxidase Gel-sol transition	[91]
**Alginate-in-alginate with palladium tetracarboxyphenylporphyrin**	Optical	Glucose	(low O_2_) Linear Range: 0.026–3.5 g/L Sensitivity: 97 ± 5.4 µs L/g (ambient O_2_) Linear Range: 0.87–3.5 g/L; Sensitivity: 7.5 ± 1.3 µs L/g	[92]
**Alginate-based microfibres with mesoporous polyester beads**	Optical	pH of epidermis	pH range: 4–9 (range for skin disorders and wounds variation)	[94]
**Titanium oxide nanotubes/alginate hydrogel**	Colorimetric assay	Biomarkers	LOD = 0.069 mM (lactate) Linear ranges: 0.1–1.0 mM LOD = 0.044 mM (glucose) Linear range: 0.1–0.8 mM	[95]
**Fluorescent chitosan**	Fluorescence	Nitrocompounds, p-nitrophenol	Nitrocompounds quench Fluorescence, LOD = 0.35–2.30 µM (2,4,6-trinitrophenol) LOD = 0.90–5.30 µM (*p*-nitrophenol)	[96]
**Extract grape skin/tara gum, cellulose nanocrystal**	Absorbance (Color change)	pH	Intensity decreases when pH increases pH range: 1–11 pH in range 1–5 λ_max_ = 528 nm. pH in range 6–10 λ_max_ = 618 nm	[74]

Abbreviations: AuNPs—gold nanoparticles; LOD—limit of detection is defined as the lowest concentration of an analyte in a sample that can be consistently detected with a stated probability (typically at 95% certainty) [111]; LOQ: limit of quantification is defined as the concentration that can be measured with a defined accuracy and precision [111]; PBS—phosphate-buffered saline, FRET—Förster resonance energy transfer, FITC—fluorescein isothiocyanate; BSA—bovine serum albumin.

#### 2.1.1. Proteins

- Collagen

**Figure 1:**
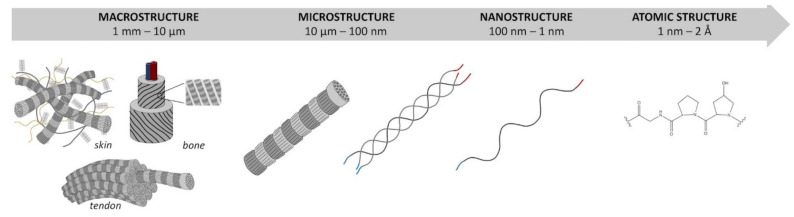
Atomic-scale of type I collagen found in skin, tendon and bones [113]. Reprinted with permission from reference [113]. Copyright 2021 from Frontiers in Bioengineering and Biotechnology.

Collagen is a protein found in the extracellular matrix of mammals’ bodies [112] that provides mechanical support against action forces to avoid repetitive plastic deformation. In addition, the different forms and orientations of fibers determine the diverse disposition of cells in the tissue [109]. The three-dimensional structure of collagen is defined by four levels (Figure 1) [113]. The primary structure consists of a sequence of -(Gly-X-Y)_n_-, where glycine represents 30% of the total amino acid content, X and Y are proline and hydroxyproline, respectively. Triplets of amino acid units define the secondary structure of the collagen. A left-handed helix, containing around 1000 amino acids, forms the third level of the organization [109]. The quaternary structure of collagen is defined by the formation of fibers, assembled by intra- and intermolecular interactions of collagen molecules [109,114]. Thanks to biocompatibility and mechanical strength, collagen has taken part in the development of novel sensors and biosensors. Despite resembling the structure and properties of native soft tissues, collagen is difficult to manipulate, and thus its derivatization before use is often required.

**Figure 2:**
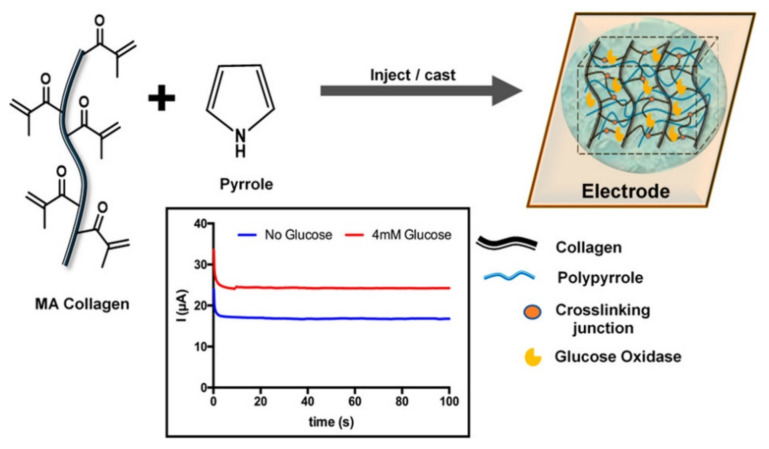
Methacrylated collagen hydrogel with polypyrrole and glucose oxidase for the detection of glucose in vitro and in tissues. Reprinted with permission from reference [80]. Copyright 2018 American Chemical Society.

Ravichandran et al. proposed a proof-of-concept of a collagen-based electroconductive sensor for glucose detection in vitro and in tissue for monitoring patients with diagnosed diabetes [80]. This device was formed by methacrylated collagen, used as a scaffold, polypyrrole representing the electroconductive polymer, and glucose oxidase, which catalyzes the oxidation of the glucose and produces the amperometric signal (Figure 2). The proposed conductive hydrogel was used to detect different concentrations of glucose in a phosphate-buffered saline solution at pH = 7.4. The sensor response of the material was assessed in porcine meat, which showed a reliable measure of glucose in this living matter at up to five days of the experiment [80].

Another example is a collagen-based biosensor proposed by Wei et al. [83]. They constructed an electrochemical biosensor for dopamine composed of collagen from grass carp skin, graphene oxide, and an aptamer. The composite collagen-graphene oxide represents the transducer, while the aptamer is the recognition element. The biosensor showed high sensitivity and a wide linear range in the determination of dopamine. In addition, it also presented good selectivity among other biomolecules, such as *L*-l-3,4-dihydroxyphenylalanine (*L*-DOPA), homovanilic acid, ascorbic acid, and tyramine. The peculiar characteristic of such a novel collagen-based sensor was the robustness in human blood serum, thanks to the biocompatibility and properties of the collagen [83].

Aronoff et al. developed imaging and targeting dual sensor for lysine oxidase based on a collagen scaffold. Lysine oxidase is important in forming crosslinks in the extracellular matrix proteins such as collagen and elastin [76]. Its inhibition can produce osteolathyrism, affecting bones and connective tissues [76,115,116]. On the other hand, the upregulation of lysine oxidase may promote the pathogenesis of fibrotic and musculoskeletal diseases and ultimately some forms of cancer [76,117]. Such a fluorescent collagen-based biosensor was highly sensitive to measure the lysine oxidase activity and interacted selectively with the endogenous aldehydes formed by lysine oxidase. The dual-modality of this biosensor allowed the targeting and imaging of extracellular collagen with high specificity and spatial resolution within in vivo and ex vivo tissues [76].

- Elastin

Elastin is a protein found in connective tissues and provides elasticity to organs. It is an insoluble polymer that presents its soluble precursor, tropoelastin, as a crosslinker [109]. Elastin is mainly formed by glycine, proline, alanine, leucine, and valine. This protein is generally organized in a repeated sequence of 3–9 amino acids which render the structure flexible and dynamic [118]. Tropoelastin and elastin-based peptides are capable to self-assemble in physiological conditions and thanks to their remarkable elasticity, biocompatibility and biodegradability, they have raised interest as scaffolds in different applications, such as 3D cell cultures, drug and gene deliveries [109]. In addition, elastin-based hydrogels have been demonstrated to be suitable for developing sensors and biosensors. 

Bandiera et al. developed a human elastin-like polypeptide fusing UnaG, an expressed protein in eel that can emit fluorescence after a high-affinity binding with bilirubin. Bilirubin is a modulator of oxidative stress and chronic inflammation processes, therefore its sensing and evaluation of concentration in biological organisms are crucial. The functionalization of elastin hydrogel with UnaG showed an affinity with bilirubin in a concentration below 100 nM, detectable using fluorescence analysis [84].

**Figure 3:**
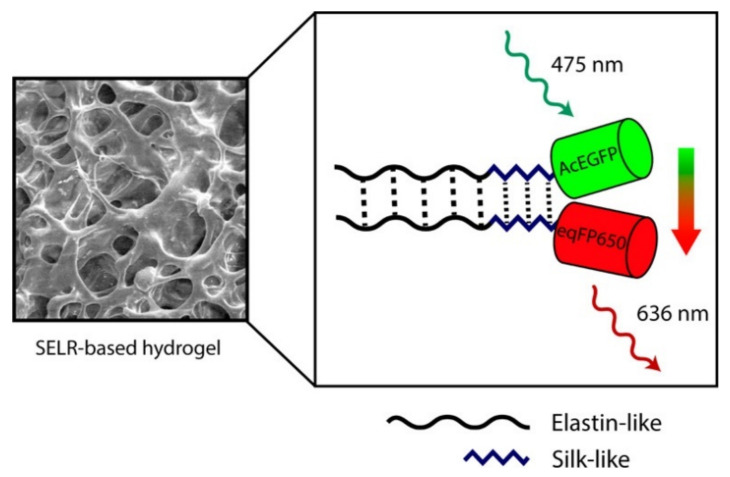
Silk/elastin-like recombinants (SELR) with fluorescent proteins hydrogel formed by silk/elastin-based network, linking two different fluorescent proteins which undergo FRET when they target biomolecules. Reprinted with permission from reference [85]. Copyright 2017 American Chemical Society.

Another example is represented by two silk/elastin-like recombinants (SELRs) with fluorescent characteristics, given by two different fluorescent proteins (FPs): AcEGFP and eqFP650 [85]. The group of Ibáñez-Fonseca studied the Förster resonance energy transfer (FRET) generated between the two silk/elastin-like recombinants. This study of FRET was performed by spectroscopy and confocal microscopy which gave information on the interactions between molecules at different concentrations (Figure 3). They found that the silk/elastin-like recombinants and fluorescent proteins can self-assemble, forming particles and hydrogels. The use of FRET as a sensory tool demonstrated by this work may increase the interest in using this material as a biosensor for different biotargets such as glucose, lipopolysaccharide, or metal ions [85].

- Gelatin

Gelatin is obtained by the denaturation of the triple helix of collagen in which, depending on the treatment, two types of gelatin are formed: type A (processed by acids) and type B (processed by alkaline solutions) [109]. These natural polymers are composed of proteins (98–99%), lacking in tryptophan and deficient in isoleucine, threonine, and methionine [119]. Hydrolyzed gelatin contains 19 amino acids which are predominately glycine (26–34%), proline (10–18%), and hydroxyproline (7–15%). Other amino acids such as alanine (8–11%), arginine (8–9%), aspartic acid (6–7%), and glutamic acid (10–12%) contribute to the structure of gelatin [120]. Such a natural polymer presents minimal immunogenicity, degradability, gel foaming, thickening, emulsifying, and foaming properties [109]. Such characteristics have been exploited in different fields, from tissue engineering to sensing/biosensing. 

**Figure 4:**
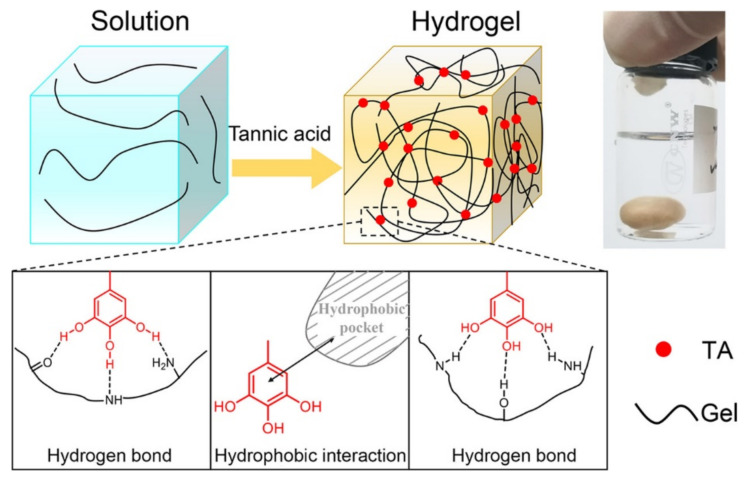
Scheme of the formation of the gelatin-tannic acid hydrogel. Tannic acid (TA) interacts with the gelatin chains through hydrogen bonds and hydrophobic interactions, forming crosslinks to the hydrogel network. Reprinted with permission from reference [87]. Copyright 2019 American Chemical Society.

Recently, a self-powered strain sensor, based on a gelatin hydrogel, was fabricated with the blending of gelatin and tannic acid (TA) (Figure 4) [87]. Such a hydrogel was able to efficiently convert chemical energy, in the form of small pressure or stretching stimuli, into electrical energy, as a voltage signal without an external power supply. Thanks to the doping of the gelatin-TA material with Ag nanowires, the conductivity of the hydrogel was effectively improved, converting efficiently pressure and stretching stimuli into a resistance signal. In addition, Zn and an air electrode were introduced into the hydrogel which was connected to a fixed resistor, obtaining a self-powered sensor system. The self-healing and self-powered abilities of the device, in addition to durability and reliability, made this hydrogel-based strain sensor a valid candidate for the fabrication of a portable and wearable electronic device [87].

Li et al. developed a gelatin methacryloyl (GelMA) hydrogel, conjugating gelatin, directly derived from the bovine skin, with methacrylic anhydride (MA). This system was able to monitor human physiological signals, pulse, and vocal cord vibration. The device was composed of layers of polydimethylsiloxane (PDMS) and GelMA (PDMS/GelMA/PDMS), used as a dielectric medium and poly(3,4-ethylenedioxythiophene) polystyrene sulfonate (PEDOT:PSS), used as an electrode. Such sensors demonstrated several benefits, including being processable in solution, their ability to reduce water evaporation, present high stability, good reproducibility, and high transparency in the visible range of the light. In addition, it showed great biocompatibility, a peculiar characteristic for potential use as a medical wearable [86]. 

**Figure 5:**
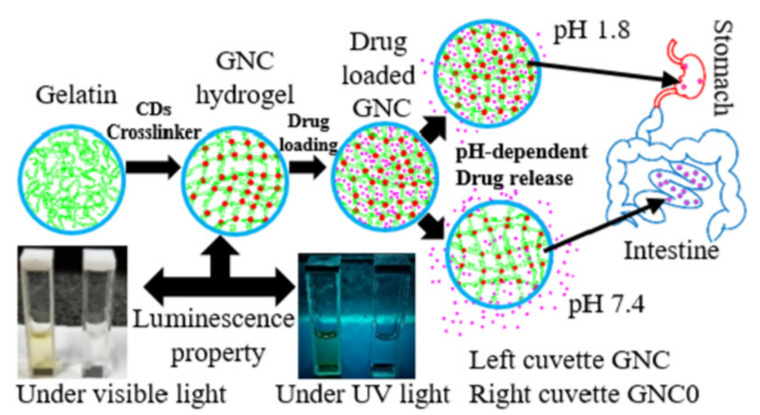
Formation of the gelatin nanocomposite crosslinked with carbon dots and loaded with a drug that is released in the gastrointestinal tract. The gelatin nanocomposite (GNC) hydrogels show fluorescence properties for biosensing and measuring the pH of the digestive system. Reprinted with permission from reference [88]. Copyright 2020 American Chemical Society.

Recently, gelatin crosslinked with carbon dots (CDs) was used as a chromophore, to form a gelatin nanocomposite (GNC) as a drug delivery system and pH sensor for the gastrointestinal tract (Figure 5) [88]. This pH-sensitive system showed remarkable photoluminescence characteristics in the near-neutral pH range of the gastrointestinal tract and also can bypass the strongly acidic environment of the stomach, releasing loaded therapeutics in the intestine. In addition, these hydrogels showed cytocompatibility and non-toxicity in the cellular environment. These results demonstrated that such GNC can be a valuable candidate for in vivo imaging, biosensing applications, and the quantitative measurement of pH in the digestive system [88].

#### 2.1.2. Polysaccharides

- Hyaluronic acid

Hyaluronic acid (HA) is found mainly in the extracellular matrix, present in the connective tissue in mammals, acting as a lubricant, as well as a signaling molecule involved in mammalian biological processes [109]. HA is synthesized by integral membrane proteins and formed by alternating units of glucuronic acid and N-acetylglucosamine which are bonded by β-(1-4) and β-(1-3) glycosidic bonds [121]. 

Modified HA hydrogels were used as a fluorescent sensor to detect hyaluronidase (HAase), an enzyme that degrades HA and plays an important role in tumor development and treatment [122]. The biosensor consisted of fluorescein isothiocyanate and gold nanoparticles coupled in the network of the HA hydrogel. The material was acting as a transducer forming a donor-acceptor pair that exploits FRET. The hydrogel is capable to interact with HAase, thanks to the crosslinks of HA derivatives, causing a FRET effect in the hydrogel, increasing the fluorescence intensity which is proportional to the concentration of the HAase. This system presented a wide response range, high sensitivity, good anti-interference, and excellent biocompatibility [90].

- Alginate

Alginic acid is a polysaccharide found in the walls of the cells of brown algae [123]. It is hydrophilic and when hydrated forms a viscous gum. Commonly, alginic acid forms salts of calcium or sodium. In the structure of alginates, β-*D*-mannuronate and α-*L*-guluronate are linked together in different sequences or blocks by either α- or β-(1-4) bonds, where the latest can form ionic bridges, conferring mechanical properties [109]. An important characteristic of alginates is the capacity to interact with multivalent cations, which results in crosslinking, to form a hydrogel that is highly water-soluble, biocompatible, and nontoxic [124].

**Figure 6:**
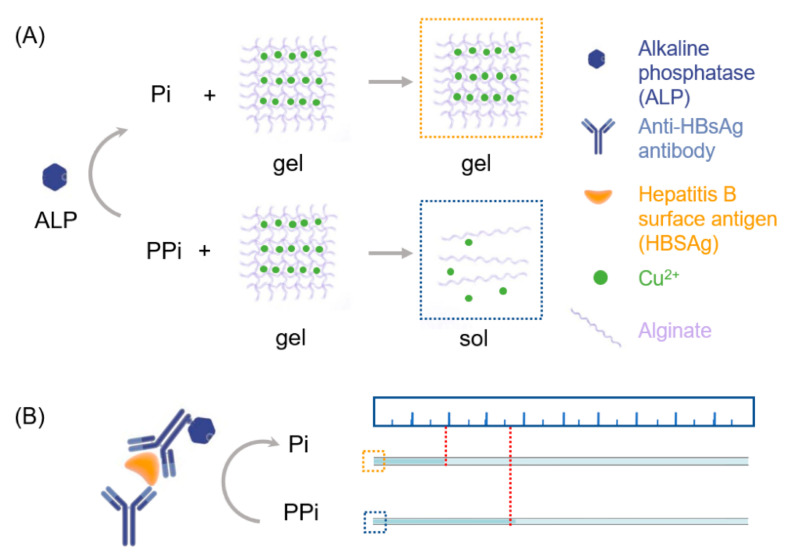
(**A**) Scheme of the quantitative immunoassay based on alginate hydrogel crosslinked with Cu^2+^ ions. (**B**) Recognition of the alkaline phosphatase (ALP)-labeled antibody. Reprinted with permission from reference [91]. Copyright 2020 MDPI.

Zheng et al. proposed an alginate hydrogel used for naked eye quantification of the immune assay. This alginate hydrogel, crosslinked by Cu^2+^ ions, is responsive to pyrophosphate. Thanks to the incorporation of the fluorescent carbon nanodots, such hydrogel can effectively quantify alkaline phosphatase, which at a high level, is responsible for a malfunction to the liver, gall bladder or bones [125]. In the presence of pyrophosphate, the material, Cu^2+^ crosslinked alginate, underwent a gel-sol transition, allowing the detection of alkaline phosphatase in serum samples with hepatitis B virus surface antigen (Figure 6). The sensitivity, linear response, and quantitative determination of alkaline phosphatase with naked eye readout demonstrated a rapid and instrument-free device that can be suitable for point-of-care tests of biomarkers for disease diagnosis, even in remote areas and temporary testing stations [91].

**Figure 7:**
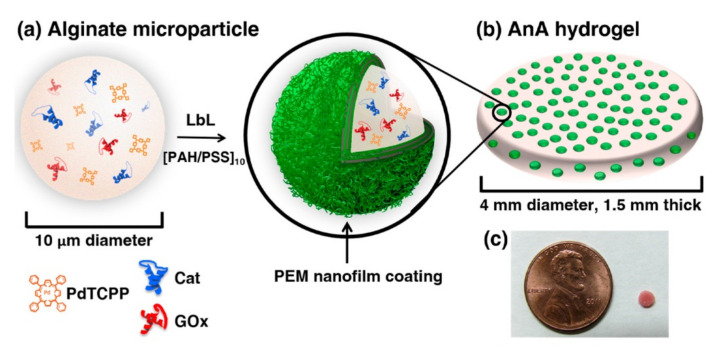
(**a**) Illustration of a microparticle of alginate consisting of Pd-*meso*-tetra(4-carboxyphenyl) porphyrin (PdTCPP), glucose oxidase (GOx) and catalase (Cat). Such microparticles are coated with 10 bilayers of poly(allylamine hydrochloride) (PAH) and poly(sodium-4-styrene sulfonate) (PSS). (**b**) AnA hydrogel with an embedded polyelectrolyte multilayer (PEM) coated alginate particles; (**c**) Photograph of alginate-in-alginate (AnA) hydrogel next to a penny. Reprinted with permission from reference [92]. Copyright 2017 MDPI.

Another example of an alginate-based biosensor is given by Bornhoeft et al. [92]. Their device consisted of an alginate-in-alginate material that embeds a nanofilm-coated phosphorescent microdomain, palladium tetracarboxyphenylporphyrin (optical indicator), glucose oxidase (model enzyme) and layer-by-layer deposited polyelectrolyte multilayers (PEMs), acting as a diffusion barrier (Figure 7). This composite hydrogel was used as a real-time optical biosensor for monitoring biomarkers, useful in precision medicine. This system responds to the changes in concentration of both glucose and oxygen due to the reactions of the glucose oxidase. When concentrations of both species decrease, the phosphorescence of the porphyrin dye is quenched by molecular oxygen. The phosphorescence intensities and lifetimes are inversely proportional to the concentration of the local oxygen. Once the concentration of oxygen decreases, the phosphorescence of the porphyrin is less quenched, therefore their lifetime increase that is in correlation with the concentration of glucose. Such a biosensor proved able to provide a controlled tuning of sensitivity and dynamic range, long-term stability, and accurate sensing at the physiological concentration range of oxygen. This approach can be useful for monitoring different oxidoreductase enzymes, crucial for chronic condition monitoring [92].

Tamayol et al. produced alginate-based microfibers incorporating luminescent mesoporous polyester beads for monitoring the pH level of the wounds on the epidermis [94]. This hydrogel was prepared in flexible patches, proving a ready and responsive point-of-care system for monitoring the progress of wound healing. The monitoring of the pH of wounds is important because it is correlated to angiogenesis, protease activity, bacterial infection, etc. In healthy skin, the pH is slightly acidic (pH = 4–6), while when the skin is wounded, the pH is alkaline. Hence, the monitoring of the pH of the epidermis is crucial to obtain useful information on the healing status. In this work, authors have chosen luminescent sensing due to its robusticity and easy-to-read system without the need for integrated electronics. Alginate microfibers gave the material flexibility, permeability, and tendency to be shaped. On the other hand, mesoporous polyester beads are the sensing part of the material, sensitive to pH changes, and give different luminescent outputs depending on the pH. During the real-time measurement of pH of wound skin, images of the hydrogel were captured with a smartphone camera, providing quantitative pH maps during the skin recovery period.

- Chitosan

Chitosan is a polysaccharide consisting of *D*-glucosamine and N-acetyl-*D*-glucosamine, connected via β-(1-4), randomly distributed in its molecular structure. It is obtained by the partial deacetylation of chitin, the main component of the exoskeleton of the arthropods [109]. Physical and mechanical features of chitosan are imparted by its molecular weight and the degree of its deacetylation. Among the main advantages of the use of chitosan hydrogels are antibacterial properties, easy sterilization, low costs, biocompatibility, and the tuning of its biodegradability due to the diverse level of deacetylation [109].

**Figure 8:**
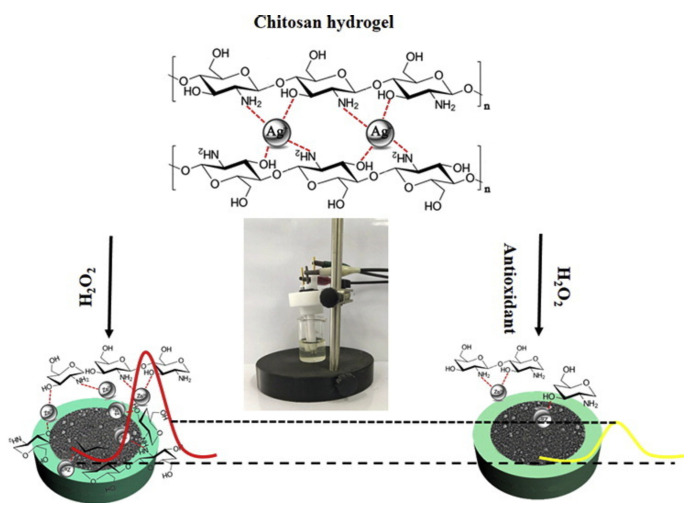
(**a**) Scheme of chitosan chelating silver ions and its redox property during the hydrogel depolymerization in the absence and presence of antioxidants. (**b**) DPV curves of the oxidation response in presence of hydrogen peroxide at various concentrations of ascorbic acid. Reprinted with permission from reference [82]. Copyrights 2017 Published by Elsevier B.V.

Fu et al. fabricated a hydrogel based on chitosan using silver ions as a crosslinking agent. The hydrogel was evaluated for sensing antioxidants. The redox properties of silver incorporated into the hydrogel decreased due to its complexation (Figure 8). In the presence of hydrogen peroxide, which easily forms hydroxyl radicals and disrupts the glucoside bonds, the redox properties of silver can be restored. This phenomenon is used as a mechanism for the evaluation of the antioxidant capacity of the hydrogel. In fact, the depolymerization of the chitosan, induced by hydroxyl radicals, releases the silver ions which can diffuse to the electrode surface and consequently give a signal correlated to the antioxidant capacity. The analytical performance of the hydrogel was evaluated using ascorbic acid as an antioxidant model. This device proved to be low cost, portable, and free of modification of the electrode [82]. 

In another example, chitosan was used as a matrix for a fluorescent sensor for nitroaromatic compounds, such as 2,4,6-trinitrophenol which have strong biological toxicity and explosive risks [96]. In this work, functionalized chitosan gels with naphthalimide, used as a fluorophore were investigated. The presence of 2,4,6-trinitrophenol and/or *p*-nitrophenol produced a notable fluorescence quenching of the hydrogels. The obtained results provided the selective and sensitive sensing of nitro compounds with ease of synthesis and low cost [96].

### 2.2. Synthetic Hydrogels

Synthetic hydrogels are man-made polymers that can present desirable mechanical properties, selective chemical reactivity and controllable molecular structure, suiting the proper fields of application [25,126]. The greatest advantage of synthetic hydrogels is the possibility to program material properties by a proper choice of building blocks [22,33]. For instance, carboxylic, hydroxylic, amino, amide, or sulfonic groups, present in the network of the hydrogel, are the driving force for the retention of water [127]. Tuning the synthetic hydrogels with such hydrophilic groups can help to introduce different content of water in the network, conferring particular properties in the polymers. The differences between natural and synthetic polymers rely on the biocompatibility, biodegradability, and the content of biologically recognizable units. Yet, natural hydrogels do not have mechanical properties, which can be an asset for the applicability in different fields of research. On the other hand, synthetic hydrogels can be tuned to obtain desired mechanical properties and other favorable properties for being employed in several applications, such as drug delivery, sensing, and self-healing materials. 

Synthetic hydrogels can display different characteristics, such as the nature of crosslinking, the type of the constituent polymers, method of preparation, physical structure, e.g., degree of crosslinking, and charge [33]. A hydrogel can be originated from either chemical or physical interactions. Crosslinking via non-covalent interactions can be reversible. Synthetic hydrogels are often formed by copolymerizing multifunctional monomers forming covalent bonds. Covalent crosslinking can be obtained either via the application of high energy [128], producing radicals in the polymer chain or via chemical reactions, such as free radical polymerization [129]. Alternatively, polymers having functional groups can be crosslinked in a post-polymerization manner using, e.g., click chemistry [130,131,132] and Schiff base [133] crosslinking. The physically crosslinked hydrogels are formed by physical interactions, such as weak interactions, e.g., hydrogen bonding, ionic interactions, van der Waals forces, etc. [134].

**Figure 9:**
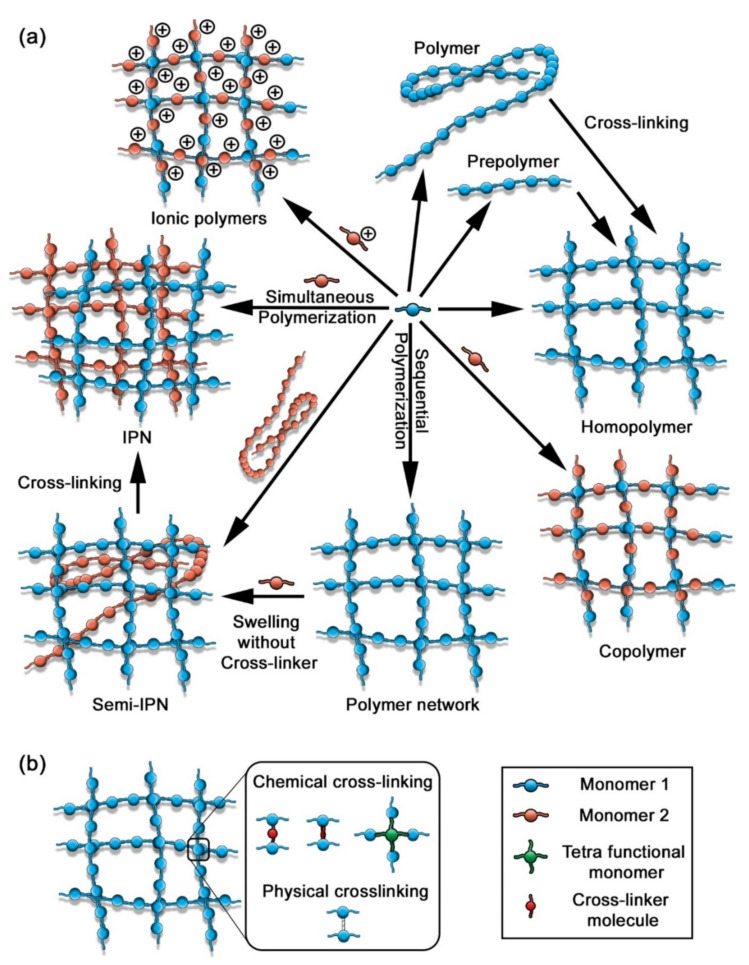
(**a**) Representation of different types of synthetic hydrogels with the methods of preparation for each type. (**b**) Different ways of crosslinking between polymer chains in a hydrogel which are between horizontal and vertical polymer chains. Reprinted with permission from reference [33]. Copyrights 2020 Wiley Periodicals LLC.

The methodology of the preparation method of hydrogels classifies them into three categories as homopolymers, copolymers, and interpenetrating networks (Figure 9). Homopolymers are constituted of only one type of monomer in their chains, while copolymers are formed by two or more kinds of monomers. These two types of polymers form only one form of a polymer chain. In contrast, the polymer chains in interpenetrating networks are different and crosslinked with each other [33]. Synthetic hydrogels can be classified based on the status of their charges which includes anionic, cationic, non-ionic and ampholytic polymers. All such ionic polymers are sensitive to pH changes, thanks to the presence of pH-sensitive groups. The preparation of the ampholytic hydrogels involves the copolymerization of cationic and anionic monomers or the embedding of a zwitterionic unit to the network [33]. Another system of classification of hydrogels is based on their crystal status and can be recognized in three groups: amorphous, crystalline, and semi-crystalline. The amorphous hydrogels present a random network structure, whereas semi-crystalline and crystalline hydrogels consist of almost or perfectly tight-packed networks [33]. 

Considering the wide range of characteristics that can be achieved by tuning in both molecular and structural levels and the ability to respond to external stimuli, such as temperature [58,65], light [66], pH [58,94], ionic strength [62,127,135], and the presence of (bio)molecules [104,136,137,138,139], synthetic hydrogels have become important materials for the design and construction of sensors and biosensors in various fields of applications. Different types of synthetic polymer-based hydrogels have been used in sensing, e.g., poly(acrylic acid) [40,105,140], poly(ethylene glycol) [36,39,141,142], poly(ethylene glycol) methacrylate [143], poly(acrylic acid-*co*-dimethylaminoethyl methacrylate) [144], poly(methyl methacrylate-*co*-methacrylic acid) [145], polyacrylamide [35,37,77,103], poly(acrylamide-*co*-acrylic acid) [146], poly(N,N-dimethylacrylamide) [78], poly(N,N-dimethylacrylamide-*co*-2-(dimethylmaleimido)N-ethyl-acrylamide-*co*-vinyl-4,4-dimethylazlactone) [102,106], poly(N-isopropylacrylamide-*co*-2-acrylamido-2-methylpropane sulfonic acid) [147], poly(vinyl alcohol) [81], poly(2-hydroxyethyl methacrylate) [75], and poly(diallyldimethyl ammonium chloride) [75]. Polymer materials are functionalized with fluorophores, chromophores, or conducting elements to enable readout using relevant detection techniques (Table 2).

**Table 2:** Synthetic hydrogel-based sensors.

Hydrogel	Sensing	Analyte	Characteristics	Ref.
**Electrochemical Methods**				
**Poly(vinyl alcohol), cellulose nanofibers and graphene**	Electrochemical	Strain	Air Linear range: 0–500% strain Variations for light-emitting diode (LED) illumination vs. different resistance	[81]
**TEMPO-oxidized cellulose in poly(acrylic acid) hydrogel, with ferric ions and polypyrrole**	Amperometry	Mechanical change (strain)	Elongation ~890% Max storage modulus: 27.1 kPa Self-healing efficiencies (electrical and mechanical): ~99.4% electro-conductibility: ~3.9 S m^−1^.	[105]
**Dendritic polyglycerol-poly(ethylene glycol) withaldehyde oxidoreductase**	Amperometry	Benzaldehyde (BA)	LOD = 0.8 µM Linear range: 0.8–400 µM Max response at pH = 4.0 Signal of BA decreases with increase of pH	[141]
**Optical Methods**				
**Polyacrylamide-phenylboronic acid**	Surface plasmon resonance, Transmittance attenuation	Glucose	PBS buffer (pH = 7.4) LOD = 0.75 mM Linear range: 0–40 mM Sensitivity 0.05–0.13 dB/mM	[35]
**Au nanoparticles-poly(ethylene glycol) diacrylate**	Absorbance, Surface plasmon resonance, refractive index	Biotin	PBS buffer (pH = 7.4) Linear range: 25Μm–0.5 mM Sensitivity 70–110 nm/RIU Fluorescence λ_max_ shift	[36]
**Polyacrylamide-DNA hydrogel containing Au nanoparticles**	Visual detection	Glucose	PBS buffer pH = 7.4 LOD = 0.44 mM Sensitivity: 1 mM Linear range: 0 to 15 mM glucose-boronic acid derivatives bind aptamer to disrupt the hydrogel, leading to the release of AuNPs	[37]
**Supramolecular poly(ethylene glycol)-poly(ε-caprolactone) with CdTe quantum dots**	Optical	pH, ions, biomolecules, chemicals, temperature	Emission of CdTe QD shifts from λ_em_ = 499 nm to λ_em_ = 549 nm	[39]
**Poly(acrylic acid)-gum tragacanth nanoparticles with CdTe quantum dots (QDs) and glucose oxidize**	Optical	Glucose	Enzyme-catalyzed oxidation of glucose produce H_2_O_2_ and quench fluorescence Linear range: 0–1 mM Blood samples media LOD-tunable	[40]
**Sodium alginate, and poly(2-hydroxyethyl methacrylate) and poly(diallyldimethyl ammonium chloride)**	Optical, Absorbance	pH	Water, acetic acid, sodium hydroxide Changing colors pH range 6.0–7.6	[75]
**Morpholino/oligonucleotide-polyacrylamide**	Optical, volumetric	ssDNA	LOD = 10 pM, PBS buffer (pH = 7.4) Gel imaged using OnePlus 5t camera, Selective swelling caused by competitive displacement of morpholino crosslinks	[77]
**Poly(N,N-dimethyl acrylamide–*co*-2-(dimethylmaleimido)-N-ethyl-acrylamide-*co*-vinyl-4,4-dimethylazlactone) (P(DMAAm-*co*-DMIAAm-*co*-VDMA)**	Surface plasmon resonance	Lysophosphatidic acid (LPA) Cancer biomarker	LOD = 2 µM Linear range: 2–30 µM Selectivity in the presence of blood components (NaCl, urea, glucose, GPA, LPC)	[102]
**Phenylboronic acid functionalized polyacrylamide**	Optical	Glucose	Operating concentration range: 0–100 mM (in PBS, pH = 7.4) Linear range: 0–50 mM Sensitivity: 11.6 µW mM^−1^ pH operating range: 6–9	[103]
**Azlactone** **terpolymer P(DMAAm-*co*-** **DMIAAm-*co*-VDMA)**	Surface plasmon resonance	Streptavidin	Linear range: 0.5–200 µM Monitoring of layer thickness of the hydrogel	[106]
**Poly(ethylene glycol) diacrylate (PEGDA)**	Fluorescence	mRNA, miRNA	LOD ≅ 6 amol (*atto*—10^−18^) (in vitro-transcribed model target). For quantification of full-length large mRNAs to small miRNAs	[108]
**Poly(acrylic acid) with immobilized urease**	Optical, volumetric	pH, urea	pH 2–12 range, 1.9–7.5 mM (urea), LOD = 40× mM (urea in blood) Change of volume and color	[140]
**Poly(ethylene glycol) methacrylate, methyl methacrylate and maleimide**	Fluorescence	Biotin-streptavidin (proteins pair model), DNA	Electrospunned nanofibers aligned into micropatterned array, that can be customized with probe that will interact with desired bioanalyte	[143]
**Poly(acrylic acid-*co*-dimethylaminoethyl methacrylate)**	pH-sensitive	Urea	LOD~1 mmol/L Linear range: 1–10 mmol/L PBS buffer pH = 7.4 Selectivity among urea, thiourea, N-methylurea and N,N,N′,N′-tetramethylurea	[144]
**Poly(vinyl alcohol) with** **carboxyfluorescein** **and poly(methyl methacrylate-*co*-methacrylic acid)** **(Eudragit S100)**	Optical	Urea	Infection-responsive coating for urinary catheters. pH > 7 dissolves the Eudragit layer, releasing the dye— visual change	[145]
**Poly(acrylamide-*co*-acrylic acid) functionalized with urease**	Particle spacing change, Debye diffraction measurement	Urea, urease inhibitor phenyl phosphorodiamidate (PPD)	LOD = 1 mM (urea) and 5.8 nM (PPD), both in water Linear range: 1–10 mM Selectivity in presence of formamide, N-methylurea, acetamide and N,N′-dimethylurea	[146]
**Poly(N-isopropylacrylamide-*co*-2-acrylamido-2-methylpropane sulfonic acid)**	Volumetric	Glucose	Operating concentration: 0–300 mg dL^−1^	[147]

Abbreviations: BSA—bovine serum albumin; FRET—Förster resonance energy transfer, FITC—fluorescein isothiocyanate; GPA—glycerophosphoric acid; LOD—limit of detection, is defined as the lowest concentration of an analyte in a sample that can be consistently detected with a stated probability (typically at 95% certainty) [111]; LOQ—limit of quantification is defined as the concentration that can be measured with a defined accuracy and precision [111]; LPC—lysophosphatidylcholine; PBS—phosphate-buffered saline.

**Figure 10:**
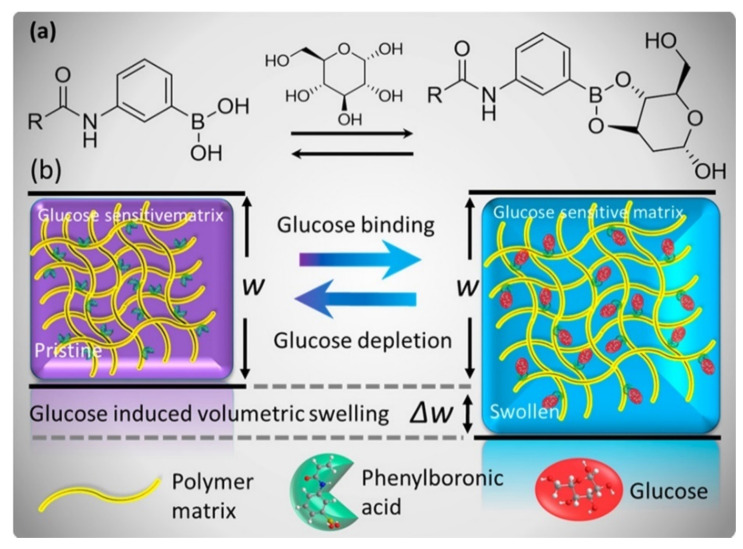
(**a**) Illustration of the binding process of glucose with the boronic acid portion, causing the swelling of the hydrogel matrix. (**b**) Illustration of the volumetric transition of the hydrogel when glucose is introduced or depleted in the matrix. Reprinted with permission from reference [103]. Copyright 2018 American Chemical Society.

Elsherif et al. proposed a synthetic glucose-sensitive hydrogel in which phenylboronic acid was incorporated into the structure of polyacrylamide hydrogel [103]. It is well-known that phenylboronic acid derivatives can reversibly bind to *cis*-diols and therefore glucose molecules [148,149]. The binding process of the phenylboronic acid moieties with glucose resulted in a change of the volume of the hydrogel matrix proportional to the concentration of glucose (Figure 10). Such a hydrogel was irradiated with a laser beam and the intensity of transmitted light generated by the sensor was measured to assess the variation of the concentrations of glucose at physiological conditions. It was demonstrated that the hydrogel can be attached to contact lenses and the intensity of the light can be measured using a smartphone. A smartphone app can convert the intensity of the incident light into values of glucose concentration. Such a sensing device allowed the creation of a low cost, rapidly fabricated, and easy detection system for glucose concentration monitoring in real-time [103].

**Figure 11:**
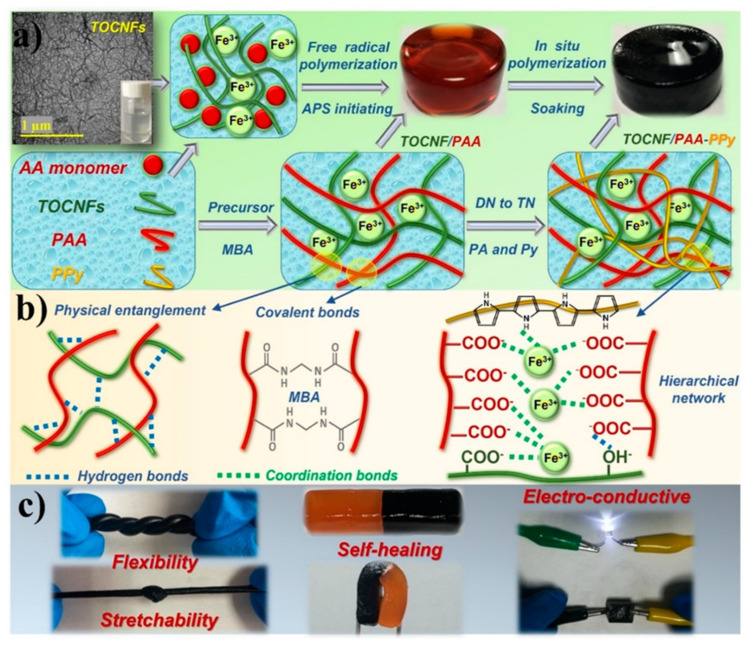
(**a**) Process of fabrication of the composite hydrogels. (**b**) Chemical and physical interactions are responsible for the formation of the triple network of the hydrogel. (**c**) Presentation of mechanical properties, self-healing characteristics and conductivity of the composite hydrogels. (Chen). Reprinted with permission from reference [105]. Copyright 2019 MDPI.

Another example of a synthetic hydrogel was introduced by Chen et al. [105]. Such a sensing hydrogel consisted of a triple-network structure based on a 2,2,6,6-tetrametylpiperidine-1-oxyl (TEMPO)-oxidized cellulose dispersed in a polyacrylic acid hydrogel with ferric ions as crosslinkers (Figure 11). Polypyrrole was incorporated in the matrix of the hydrogel as a conductive network element. Thanks to the interlocked structure created by hydrogen bonds, ionic coordination interactions, and physical entanglements, the composite hydrogels showed a homogeneous structure, high mechanical stretchability, high viscoelasticity, and ability to self-heal. The hydrogel sensor was able to detect both small and large scale human movements with a sensitive, fast, and stable current response. This result demonstrated that such a sensing hydrogel can be promising for applications as a wearable electronic device [105].

**Figure 12:**
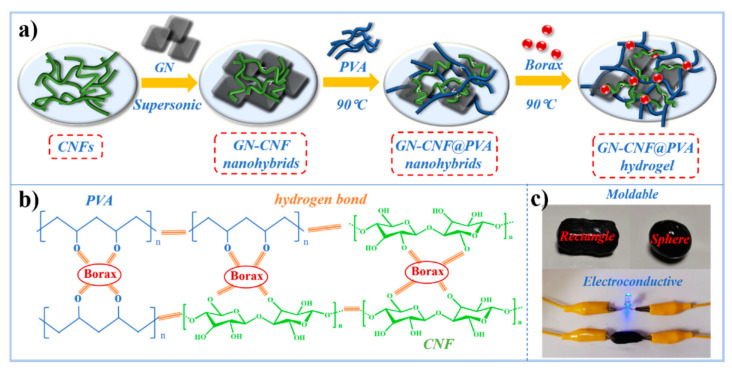
(**a**) Schematic representation of the preparation of graphene-cellulose nanofibers with poly(vinyl alcohol) (GN-CNF@PVA) hydrogels; (**b**) representation of the mechanism of the formation of the hydrogel network with borax; (**c**) illustration of malleability and electroconductivity of the hydrogels. Reprinted with permission from reference [81]. Copyright 2019 MDPI.

Recently, Zheng et al. proposed a hybrid hydrogel for strain sensing composed of cellulose nanofibers (CNF) and graphene (GN), which incorporates poly(vinyl alcohol) (PVA) and borax, used as a crosslinker (Figure 12) [81]. In this work, cellulose in the form of nanofibers was introduced in the hydrogel matrix to improve mechanical properties and strong interactions into the network. The presence of PVA confers crosslinks to the hydrogel network. Thanks to blending with the nanocellulose, the hydrogel shows intrinsic hydrophilicity, biocompatibility, biodegradability, and high crystallinity. Graphene was introduced in the hydrogel matrix to confer mechanical strength and electrical conductivity. Cellulose nanofibers promoted graphene dispersion in the hydrogel. The formation of the hydrogel composite was achieved thanks to the graphene-cellulose nanocomplexes, the hydrogen bonding system created with PVA, and the crosslinks formed by borax resulted in an electroconductive, elastic and mechanically strong material. This material was tested to monitor human movements in a wearable device, which demonstrated excellent sensitivity, repeatability, and stability in the signals. The hydrogel can be a promising strain sensor for intelligent wearable devices [81].

Recently, the work of Kertkal et al. showed the preparation of two types of hybrid hydrogels [75]. The first hybrid hydrogel consisted of a blend of a natural polymer, sodium alginate, and synthetic polymers poly(2-hydroxyethyl methacrylate) (PHEMA) and poly(diallyldimethyl ammonium chloride) (PDADMAC). The other hybrid hydrogel was obtained by mixing the synthetic polymers with inorganic silica nanoparticles. Bromothymol blue was added in both types of hydrogels as a pH indicator due to its color changes, depending on the acidic/basic environment. Such hybrid hydrogel systems can be used as a chemical sensor for monitoring pH changes in different application fields, such as the food industry, environment, and urine overflowing in diapers. The presence of PDADMAC, sodium alginate, and silica influenced the optical and swelling properties of the hydrogels mixed with bromothymol blue. In addition, the content of PDADMAC affected the brightness of the colors of the hydrogels. Such a system proved to be an easy and sensitive tool for the monitoring of pH changes in different conditions. 

**Figure 13:**
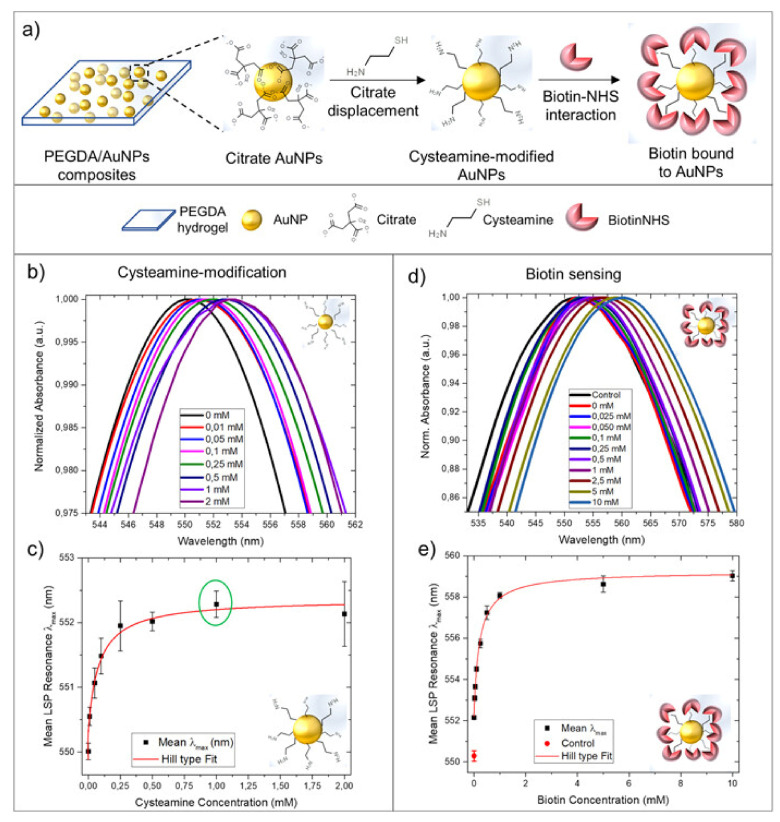
Biotin detection with poly(ethylene glycol)-based embedded with gold nanoparticles (PEGDA/AuNPs). (**a**) The preparation process of hydrogel PEGDA/AuNPs, modification of gel trapped nanoparticles involves the citrate displacement with a cysteamine modification of Au and the biotin grafting on the available amino groups of cysteamine. (**b**) Absorbance spectra of the hydrogel as a function of cysteamine concentration. (**c**) Shift of λ_max_ as a function of the cysteamine concentrations from 0:01 to 2 mM; (**d**) Absorbance spectra of the hydrogel as a function of biotin concentration ranging from 25 μM to10 mM. (**e**) Shift of λ_max_ as a function of the biotin concentration (from 25μM to 10 mM). Reprinted with permission from [36]. Copyright 2021 AIP Publishing.

Poly(ethylene glycol)-based hydrogels are popular materials for sensing application [36,39,141]. For example, poly(ethylene glycol) diacrylate with embedded gold nanoparticles was used as an optical sensor for biotin [36]. The gel environment provided the stability of the trapped colloidal Au nanoparticles solution compared to water solution, presenting an opportunity to use the plasmonic effect as a biotin indicating signal. Biotin, used as a model molecule, has been captured and optically detected with a transmission mode customized setup using cysteamine modified Au nanoparticles (Figure 13). The developed device can be used for the detection of other types of biomolecules in water.

Xie et al. developed a preliminary fluorescent supramolecular hydrogel sensor consisting of semiconductive CdTe quantum dots (QDs), stabilized with an amphiphilic block copolymer, made of mercaptan derivatized poly(ethylene glycol)-poly(ε-caprolactone) [39]. The self-assembly of the supramolecular hydrogel was achieved thanks to the interaction between the amphiphilic block copolymer and the QD. In addition, α-cyclodextrin (α-CD) was added to the network of the hydrogel. Changing the amounts of the block copolymer, α-CD, or QDs modulates the gelation kinetics, the mechanical strength of the hydrogel, and most importantly, changes the fluorescence characteristics of the hydrogel. In addition, the fluorescence behaviour of the supramolecular composite can also be changed by external factors, such as temperature and pH. Such features allowed the development of a promising supramolecular hydrogel, biocompatible and responsive to external stimuli, useful as an optical biosensor [39].

## 3. Foldamers in Sensing

Foldamers are synthetic molecules that adopt a conformationally ordered state in solution, similarly to biopolymers such as proteins or nucleic acids [150,151,152]. Therefore, foldamers gained popularity in light of the possibility of designing molecules in such a way that they have defined functionalities similar to natural macromolecules [153]. Moreover, the enormous number of properties possessed by biopolymers is encoded by relatively small “alphabets” of monomers, namely 20 amino acids in proteins and four nucleobases in nucleic acids. The great potential inherent in foldamers results from the possibility of extending the alphabet with abiotic monomers. Furthermore, the development of precision polymer chemistry methods [154,155,156] leading to sequence-defined macromolecules provides opportunities for new types of foldamers based on an abiotic backbone [157,158,159,160,161].

The tendency of foldamers to form diverse secondary structures makes them very interesting objects in material sciences [162]. They can be exploited as peptidomimetics [43], e.g., an additional methylene unit in the β-peptides backbone generates a new stereogenic center and substitution position. This makes them unrecognizable by traditional proteases, which in turn means that they are intrinsically resistant to degradation [42,163]. They can form 8-, 10-, 12-, and 14-helices, depending on the monomer structure guided by the formation of the hydrogen bonds, responsible for structure formation [150,164,165]. The secondary structure of foldamers can be fitted to particular molecules, forming capsules to size [166,167]. The improved structural stability of foldamers can be achieved by intramolecular side-chain crosslinking, providing good material stability [168]. Among foldamers, we can identify amide-based macromolecules (α-γ-peptides [169], peptoids [170,171]), oligoureas [172], and oligoaryls [173]. The large library of structural motifs of foldamers enables the design of countless functions and applications [174,175,176], including sensing [151,177]. Thanks to the structural control of foldamers, they can be designed to bind complementary guests [178], e.g., cations [179,180,181,182,183,184,185,186], anions [186,187,188,189,190,191,192], or non-charged molecules [164,193,194,195,196,197,198,199,200,201,202,203,204,205,206,207,208,209]. They have been applied in the detection of metal ions [210,211,212,213], explosives [214], biomarkers [215], pH [216,217], membrane curvature [218], and fructose [198] (Table 3).

Liu et al. proposed a hexameric oligophenol foldamer to detect Cu^2+^ ions [210]. They confirmed that metal ions can induce folding by stabilizing polymer conformation. The linear, a more fluorescent form of the oligomer in the presence of ions folds into a curved structure of smaller fluorescence intensity. The copper-induced change in intensity makes it possible to detect bound metals and even selectively detect copper ions. This approach is promising in the context of the selective detection of metal ions using analogous compounds, in which the change of conformation occurs under the influence of particular ions [210].

Davis et al. constructed water-soluble foldamers for non-Faradaic capacitive anion detection [211]. In this approach, they have synthesized halogen (XB) and hydrogen bonding (HB) anion receptors. The addition of perrhenate anions, iodide, and thiocyanate induced an increase in the capacitance of the material. Interestingly, the exposure of XB and HB to perchlorate, nitrate, or bromide did not generate any notable change in capacitance, indicating the selective binding of charge-diffuse anions by the foldamer. The detection limit for XB was the lowest for iodide ions, while the HB foldamer turned out to be about three times less sensitive, emphasizing the advantage of the halogen-bonding interface. This new strategy should enable the detection of each anion using the suitable anion receptor [211]. 

Fu et al. described a chromogenic sensor based on squaraine foldamer controlled by Ca^2+^ ions [219]. When the sensor is bound with calcium ions, it allows the binding of oxalate and its detection with the “naked eye” based on changes in fluorescence. Emission and absorption spectra of the foldamer itself and with the increasing amount of calcium ions differs. Two dye molecules bind one calcium ion creating a sandwich dimer with decreased intensity of absorption at 635 nm and a hypsochromic band formed at 565 nm, quenching the fluorescence. Thus, the observed color of the solution changed from cyan to blue. In the presence of oxalate ions, which bind calcium, the quenched fluorescence can be restored. The removal of calcium ions from the complex and the unfolding of the polymer is confirmed by the increase in the maximum absorption at 640 nm and emission at 660 nm. Other metal ions, such as Li^+^, K^+^, Na^+^, Mg^2+^, Ba^2+^, or Sr^2+^, caused no changes in emission and absorption. The material was used as a switchable fluorescent probe for the detection of oxalate ions. This detection system may find application in food safety assessment, clinical diagnosis of irregular oxalate levels, or the detection of calcium-binding anions present in the human body [219]. 

**Figure 14:**
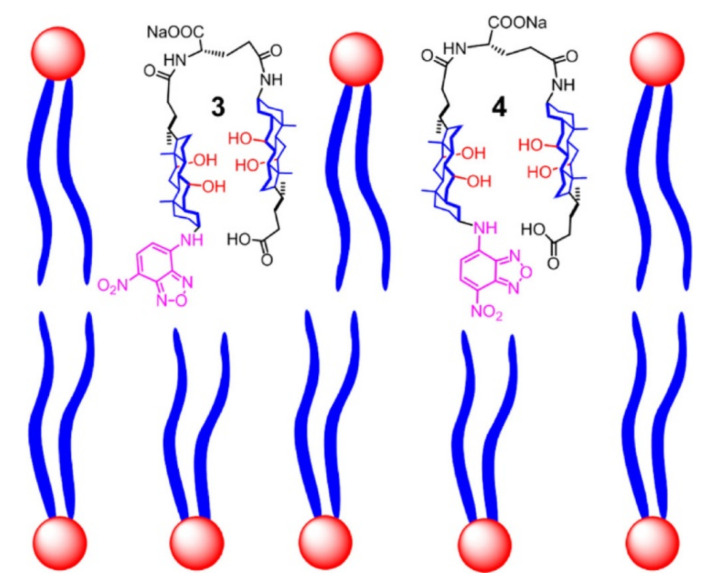
Schematic representation of possible conformations of the foldamer **3** and **4** in the lipid bilayer. The fact that **3** emitted at a significantly lower wavelength than **4** suggests that the former was located in a more hydrophobic microenvironment than the latter. Reprinted with permission from reference [218]. Copyright 2015 American Chemical Society.

Gunasekara and Zhao demonstrated a *bis*-cholate foldamer applied as an effective membrane-curvature sensor [218]. They investigated four foldamers in the presence of liposomes of different sizes as model molecules, i.e., different membrane curvatures, using fluorescence detection. The sensor with a non-ionic fluorescent label located on the α-face of the attached cholate revealed the most advantageous properties. The foldamer displayed the strongest emission enhancement, monotonous response to lipid curvatures, and the strongest binding of lipid membranes. The change in the properties was related to the foldamer conformation (Figure 14). The foldamer-based material is characterized by a much simpler bis-cholate synthesis compared to the classically used sensors (proteins or amphipathic α-helices), which makes them useful as biosensors [218]. 

Another example of a foldamer for selective sensing is a macrocycle-based dinuclear foldamer constructed by Hossain et al. [217]. The dinuclear copper(II) complex in the presence of eosin (EY), a fluorescent dye, was investigated as a system for the detection of molecules. The fluorescence intensity of EY continuously decreased upon the increasing addition of the complex to the solution of the dye, resulting in an almost absolute quenching of the emission. The created adduct was tested in the detection of inorganic halides, oxoanions, and carboxylates in water at neutral pH. The foldamer complex was the most effective in detecting citrate followed by oxalate, glutamate and phosphate. These anions caused the greatest restoration of fluorescence by displacing the dye from the complex. The optical properties of the probe were also analyzed and a color change from magenta to pale orange was observed after the addition of citrate, tartrate, and phosphate. Cytotoxicity tests of the EY complex on human foreskin fibroblast cells confirmed the biocompatibility of the compound up to a concentration of 100 µM. Therefore, the sensor can be used to detect citrate ions in biological systems [217].

Wolf et al. developed an oligo(phenylene)ethynylene foldamer with peripheral bis(trifluoromethyl)phenylurea units for the detection of chiral carboxylic acids [216]. The effect of this chiroptic sensor is based on the CD measurement of analyte samples in the presence of base in CHCl_3_ and acetone. The characteristic CD signals occur due to the formation of a hydrogen bond complex between the foldamer and the enantiomeric form of the analyte. The probe was tested for the quantitative analysis of non-racemic tartaric acid mixtures and the enantiomeric excess error was determined at the level of 0.2–6.4%. Hence, this sensor can be successfully applied on a larger scale because of its simplicity and short time of analysis adaptable to high-throughput screening technology [216]. 

Martinek et al. designed an ELISA-foldamer test for sensing Aβ-oligomers which plays a key role in the pathogenesis of Alzheimer’s disease [215]. The scientists have created a sandwich test consisting of a biotin-labeled foldamer immobilized on streptavidin-coated plates. The foldamer captured Aβ-oligomers, which were optically detected by a monoclonal mouse antibody and an anti-mouse secondary antibody conjugated to horseradish peroxidase. This optimized ELISA-foldamer was sensitive to Aβ-oligomers in the picomolar range. Studies have confirmed its selectivity for Aβ surface patterns transiently present during the ongoing aggregation process. These results imply that protein mimicking foldamers could be useful agents in biosensors and affinity assays [215].

**Figure 15:**
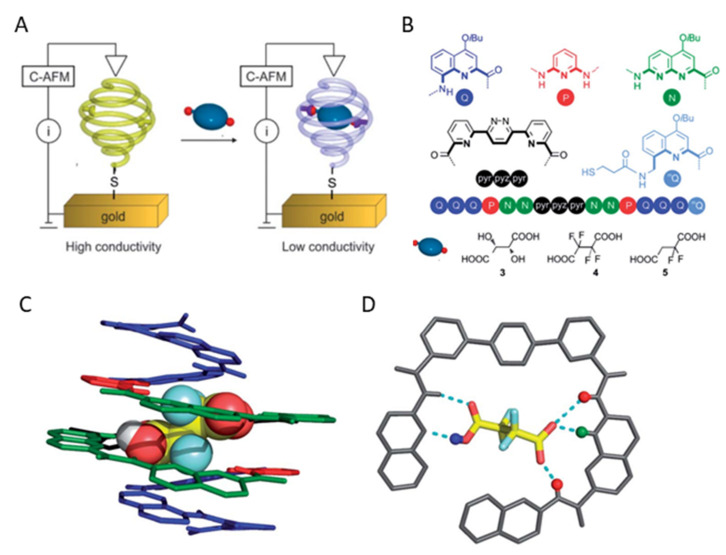
(**A**) Foldamer grafted to a gold surface constitute a probe for detection of diacids. Conductive AFM assesses the effect on conductivity upon protonation of the foldamer backbone via the recognition of an acidic guest. (**B**) Chemical structures of foldamer building blocks. (**C**) The guest is shown in foldamer-filling representation. (**D**) Top view of the central part of the complex. Reprinted with permission from reference [220]. Copyright 2021 the Royal Society of Chemistry.

A sensor-based on selective binding of dicarboxylic acids to an oligoamide foldamer was recently reported by Huc et al. [220] (Figure 15). The structure of foldamer was designed to recognize acids through multiple non-covalent interactions. The involved binding forces were a combination of electrostatic protonation/deprotonation, hydrogen bonding, and geometrical constraints. The foldamer with a thiol anchoring group was immobilized on a gold surface and evaluated for sensing of *L*-tartaric acid, tetrafluorosuccinic acid, and 2,2-difluorosuccinic acid. The foldamers underwent fast complexation of 2,2-difluorosuccinic acid with deprotonation of one of the two carboxylic acid groups that showed a significant difference (about 80-fold) in the charge transport. The conductivity change was measured by AFM. Guest binding to an immobilized foldamer changes its electrical properties, which is an important step towards the de novo design of electronic sensors that exploit molecular recognition in signal transduction.

Zhang et al. demonstrated tetraphenylethylene foldamers with double hairpin turn linkers capable of detecting 0.88 fg TNT vapors per mL of air [214]. As the molecular weight increased, the foldamers began to aggregate into hollow structures sensitive to the TNT-contaminated air. The TNT molecules bind to oligomer via N–π interactions and hydrogen bonds between TNT nitro groups and aromatic rings. Thus, foldamer fluorescence is quenched. This discovery will lead to the development of foldamers for the detection of other explosives with nitro groups, such as nitramines and nitroalkanes [214].

Zhang et al. described sensors based on gel foldamers [212]. They developed a tri-pillar[5]arene (FSOF) based xerogel foldamer for the detection of ions such as Fe^3+^, Hg^2+^, and Cr^3+^. The sensors show aggregation-induced emissions that display fluorescence quenching in the presence of metal ions. The formation of the gel allows metal ions to be separated from their aqueous solution by absorption (for FSOF at the level of 92.39–99.99%), while the addition of ions to FSOF made it possible to construct a foldamer (MSOF) for the detection of CN^−^ and H_2_PO_4_^−^ and their separation. The developed foldamers allow for ultra-sensitive multi-analyte detection and highly effective separation of ions [212].

## 4. Conclusions and Future Outlook

Polymers are attractive materials for next-generation sensory materials. They offer a wide range of available monomers that provide various properties. Polymer materials are evolving with the development of precision synthesis tools. Accessible, easy approaches for the preparation and modification of macromolecules enable improved control of their properties. In turn, the facile modulation of the properties allows the fine-tuning of the material functionalities in order to fabricate refined sensors, meeting the market needs. Current requirements for sensors are low cost, feasibility, short analysis time, as well as high sensitivity and selectivity. It is challenging to acquire all these parameters at the same time. Having a high sensitivity and selectivity while maintaining simple use is not an easy task. Therefore, sensory materials are mostly designed for cheap and accessible detection techniques, usually amperometric or optical, that eliminate the barrier of having sophisticated apparatuses. The use of a smartphone as a tool for the detection and quantification of substances is becoming more and more common. The presented examples show that in order to achieve the appropriate detection parameters, high sensitivity and selectivity of the probe material are required. To reach satisfactory sensing parameters, we have to adjust the chemical structure of the material and its morphology in the nanoscale. Proper adaptation of the polymer material to the analyte is crucial for achieving high sensing parameters. The challenge is a proper design of recognition elements to have a strong and selective affinity to an analyte. This can be achieved by constraining the macromolecule shape by crosslinking or monomer sequence control. Therefore, gels and foldamers are attracting significant interest as sensor probe materials. Foldamers characterized by specific spatial structures and cavities that can be fitted to target molecules are of particular interest. It is expected that foldamers can display similar receptor functionalities as natural biopolymers. Yet, the rational foldamer structure design is not a trivial task. Currently, little is known about the structure–property relationship of abiotic macromolecules. Even for natural proteins, built from a finite number of 20 amino acids, being the object of extensive studies over the last 50 years, the sequence–property relationship is not fully understood. However, significant progress has been made recently with the support of artificial intelligence tools. Computational methods are highly abundant in biological science, and they should be introduced to material science to accelerate the development of the field.

To fulfil current requirements for sensory materials, we should learn how to mimic natural receptors present in living systems. The expectations can be met by inducing relevant shapes and functional groups into macromolecules that will be involved in specific binding. The foldamers are interesting candidates to display desired properties. By controlling the monomer sequence, i.e., the primary structure of a macromolecule, we can gain control over a secondary structure, as it is observed for natural proteins. This approach offers lots of possibilities in sensor design. The proper choice of monomers and their alignment may form a binding cavity to fulfil the selectivity criteria, since any other molecule of a different shape will simply not fit. The fit of the material to the shape of the analytes has already been investigated for molecular imprinting technology. The molecularly imprinted polymers are synthesized in the presence of an analyte that is washed out in further steps. When the molecule is released from the polymer, it leaves its cavity template printed into the polymer matrix. However, it is accompanied by selectivity restrictions related to the limited structural precision of the polymerization process, especially pronounced for sensing macromolecules. The existing limitations could be overcome by using sequence-defined polymers. They merge features from two worlds: biological structure precision and a large library of synthetic building blocks representing a wide range of properties. Sequence-defined polymers, thanks to full structure control, could be programmed and precisely engineered to obtain desirable properties of sensory materials.

## Figures and Tables

**Table 3:** Foldamer-based sensors.

Foldamer	Sensing	Analyte	Characteristics	Ref.
**Methionine-cholate hexamer**	Fluorescence	Hg^2+^	Organic solvents Linear range 0–0.24 μM Dansyl fluorescent dye	[182]
**Hexameric oligophenol**	Fluorescence	Cu^2+^	THF with 1% DMSO solution 90% fluorescence quenching λ_ex_ = 351 nm	[210]
**Tetratriazole**	Impedance-derived capacitance spectroscopy	ReO_4_^−^ I^−^ SCN^−^	LOD: 28 µM (XB), 80 µM (HB) 14 µM (XB), 47 µM (HB) 42 µM (XB), 113 µM (HB) H_2_O with 100 mM NaCl solution	[211]
**Tri-pillar[5]arene (FSOF) FSOF-Cr** **FSOF-Fe**	Fluorescence	Ions	LOD: 1.18 nM Fe^3^ 1.86 nM Cr^3+^ 0.94 nM Hg^2+^ 1.78 nM H_2_PO^4−^ 2.12 nM CN^−^	[212]
**Dithiocarbamate**	Fluorescence	Hg^2+^	LOD = 3 10^−13^ M λ_ex_ = 278 nm, λ_em_ = 326 nm and 339 nm. Selectivity in presence of other ions	[213]
**Tetraphenylethylene with hairpin linkers**	Fluorescence	2,4,6-trinitrotoluene (TNT)	LOD = 0.88 fg/L of air Fluorescence quenching with increasing of TNT	[214]
**β-peptide**	Immunoassay	Aβ-oligomers	LOD = 5 pM Linear range: 10–500 pM	[215]
***Bis*** **(urea)oligo(phenylene)ethylene**	Circular dichroism	Carboxylic acids	λ_max_ = 370 nm Linear correlation for CD amplitude: −100–100 %ee %ee of tartaric acid: 0.2–6.4 %error	[216]
**Dinuclear macrocycle-based copper complex**	Fluorescence, colorimetry	Citrate	LOD = 0.45 ± 0.02 µM in water (pH = 7) Linear range: 1.25–8.60 µM λ_ex_ = 470 nm, λ_em_ = 536 nm	[217]
***Bis*** **-cholate**	Fluorescence	Membrane curvature	PBS buffer (pH = 7.4) λ_exc_ = 470 nm λ_em_ = 521–550 nm The binding affinity defined as K_p_ (10^3^ M^−1^), max. = 77 ± 10 4-fold when liposome size change	[218]
**Thioether linked biochromatic squarine**	Fluorescence	Oxalate	LOD = 5.2 nM Before binding: λ_abs_ = 627–622 nm, λ_em_ = 657–687 nm After binding: Decrease λ_abs_ = 635 nm, shifted band λ_abs_ = 565 nm Decrease λ_em_ = 652 nm	[219]
**Aromatic oligoamide**	Conductivity	*L*-tartaric acid tetrafluorosuccinic acid, 2,2-difluorosuccinic acid	80-fold variation of its conductance upon binding was detected by AFM	[220]

Abbreviations: AFM—atomic force microscopy; BSA—bovine serum albumin; DMSO—dimethyl sulfoxide; K_p_: apparent molar partition coefficient; LOD—limit of detection, is defined as the lowest concentration of an analyte in a sample that can be consistently detected with a stated probability (typically at 95% certainty) [111]; PBS—phosphate-buffered saline, FRET—Förster resonance energy transfer, FITC—fluorescein isothiocyanate;. THF: tetrahydrofuran.
